# Examining School and Neighborhood Effects of Socioeconomic Status on Childhood Obesity in the U.S.

**DOI:** 10.3390/ijerph19105831

**Published:** 2022-05-10

**Authors:** Christian E. Vazquez, Megan J. McBride, Katherine E. Hess, Catherine Cubbin, Sarah Kate Bearman, Esther J. Calzada

**Affiliations:** 1 School of Social Work, The University of Texas at Arlington, Arlington, TX 76019, USA; 2 Department of Educational Psychology, College of Education, The University of Texas at Austin, Austin, TX 78712, USA; meganmcbride@utexas.edu (M.J.M.); kaelhess@utexas.edu (K.E.H.); skbearman@austin.utexas.edu (S.K.B.); 3 Steve Hicks School of Social Work, The University of Texas at Austin, Austin, TX 78712, USA; ccubbin@austin.utexas.edu (C.C.); esther.calzada@austin.utexas.edu (E.J.C.)

**Keywords:** Latino/as, childhood obesity, neighborhoods, socioeconomic status

## Abstract

Obesity amongst Kindergartners in Texas is above the national average, particularly among Hispanic students. Research on the impact of school and neighborhood-level SES on obesity in childhood using multilevel models is lacking. Survey data were collected from Hispanic caregivers of pre-kindergarten students in Fall 2019 (*n* = 237). Students were clustered in thirty-two neighborhoods and twelve schools. The dependent variable was the child’s body mass index z-score (BMIz). Covariates included the child’s sex, primary caregiver’s marital status, education level, relationship to the child, and family income. Level-two variables included neighborhood poverty and school SES. CTableross-classified multilevel linear regression models were conducted to examine the unique associations of neighborhood poverty and school SES with individual student BMIz, and how they interact. Twenty-four percent of students were classified as overweight, and five percent were classified as obese. The models resulted in a significant association between school SES and BMIz (*B* = −0.13; *SE* = 0.06; *p* < 0.05) and between neighborhood poverty and BMIz (*B* = −1.41; *SE* = 0.49; *p* < 0.01). Individual students’ BMIz decreased as school SES increased and decreased as neighborhood poverty increased. Neighborhood poverty and school SES appear to play a role in the development of obesity in childhood, although in differing directions.

## 1. Introduction

Hispanic children experience obesity at disproportionately higher rates compared to White and Asian children [1]. Early childhood (ages 2–4 years) obesity is approximately 16% in Texas, considered one of the top 10 worst prevalence rates in the United States [1]. Even more specific to the current study’s population, the prevalence of obesity amongst Kindergartners in South Texas is estimated to be around 18% [2]. Over half of South Texas’ population identifies as Hispanic, and this number continues to rise [3]. Further, children from families experiencing poverty are placed at increased risk for early childhood obesity, and in Texas, around 20% of Hispanic families were experiencing poverty as of 2019, compared to less than 10% of their White counterparts [1,4].

When considering these documented disparities in obesity, in addition to a growing Hispanic population, it is imperative to examine factors beyond the individual level that may promote a public health approach to addressing disparities of obesity in early childhood. Targeting obesity in early childhood allows for the prevention of obesity conceivably, and prevention of obesity in childhood reduces the risk of chronic disease [5], as well as economic costs in adulthood associated with early childhood obesity (e.g., increased health expenditures) [6]. A recent review documented that research of various designs (e.g., longitudinal, intervention) has identified early childhood as the most important time to prevent or reduce obesity. Early childhood is a critical developmental period physiologically and in regard to the development of healthy lifestyle habits [7].

### 1.1. Individual/Family Level Factors

Research on the impact of individual/family socioeconomic status (SES) on BMI and behaviors related to BMI (e.g., physical activity and healthy eating) is abundant and largely consistent. In the United States, higher levels of individual/family SES have been consistently associated with lower rates of obesity in childhood [7]. While most children in the United States—irrespective of family SES—fail to meet public health recommended physical activity levels and fruit and vegetable consumptions levels, children from families with lower SES still tend to have lower levels of these healthy behaviors compared to children from families with higher SES [8,9,10,11,12]. Additionally, at the family level, caregiver marital status, as well as caregiver relationship to the child, are also associated with overweight/obesity. A recent systematic review examining the prevalence of obesity for children living in single-parent households found higher BMIs in children of single-parent households as compared to multi-parent households [13]. This was most prevalent among girls and Black children [13]. In Pulgaron et al.’s [14] study, grandparents served a protective role on BMI z-scores for the youth of Hispanic descent. However, less is known about how factors beyond the individual/family level impact BMI.

### 1.2. School Level Factors

School-related factors are important for understanding childhood obesity, especially given the connection between school resources and nutrition that may impact weight status [15,16]. Several studies examining school-level factors and obesity focused on the relationship between public/private status and the percent of students eligible for the national free or reduced-price lunch (FRPL) program. For example, Li and Hooker’s cross-sectional study [17] found that regardless of household socioeconomic status, children attending public schools had higher BMI than those attending private schools, and eligibility for free or reduced-cost lunch or breakfast programs at public schools was positively correlated with children’s BMI. Williams et al.’s [18] systematic review of studies examining the relationship between the free or reduced-price breakfast and lunch programs and obesity suggests it is unclear if these programs are beneficial for children’s health-related to obesity. Findings using different measures of SES are difficult to compare; however, this adjacent area of the literature is relevant to the current study for two reasons. First, all the students at the schools involved in this study are eligible to participate in the free or reduced-price breakfast and lunch program. Second, all of the schools included in the present study are public schools. These studies using proxies of school SES help provide insights for the current study, which utilizes a different proxy for school SES based on aggregate family income.

### 1.3. Neighborhood Level Factors

Findings from cross-sectional studies provide some insights on the relationship between neighborhood SES and obesity in childhood and behaviors related to obesity—physical activity and healthy eating. Singh et al. [19] found that the odds of a child being obese or overweight were 20–60% higher among children in neighborhoods with the most unfavorable social conditions (e.g., poor housing) in comparison to children not facing said conditions. Further, these effects were larger for younger children compared with older children [19]. Kim and Cubbin’s [20] study of neighborhood poverty and neighborhood income inequality found that poor children living in non-poor and unequal neighborhoods (i.e., census tracts below the median poverty rate (<10.2%) and at or above the median GINI index (>0.35)) had the highest odds of reporting insufficient physical activity than poor children in other types of neighborhoods (i.e., non-poor and equal, poor and unequal, poor and equal). Their results suggest that neighborhood economic context presents a social barrier to healthy behaviors among poor children. Other studies have reported neighborhood SES as being positively associated with physical activity and fruit and vegetable intake and that children from lower SES neighborhoods tend to have higher BMIs [11,21,22,23]. Lee and Cubbin [24] found that low neighborhood SES was independently associated with poorer dietary habits, but it was not associated with physical activity. Conversely, Kimbro et al. [25] found that neighborhood physical disorder (e.g., physical condition of the street and surrounding buildings) was associated with both more outdoor play but also more television watching. Similar to the literature surrounding school SES and obesity, there is a lack of standardization in measuring neighborhood SES, and it also tends to be measured with proxies. However, these studies informed the development of the current study, which examines neighborhood poverty and obesity.

### 1.4. The Gap in the Literature

Less is known about which level (or levels, e.g., individual, school, or neighborhood) of contextual factors, such as SES, influences healthy weight among preschool-aged children. Rossen [26] utilized multilevel models to examine various levels of SES and found that there was a significant interaction between individual-level SES and neighborhood deprivation, where higher individual-level income was protective for children living in low-deprivation neighborhoods but not for children who lived in high-deprivation areas. Additionally, area deprivation was associated with higher odds of obesity, but only among children who were above the poverty threshold [26]. Beyond Rossen’s [26] study, these relationships have typically only been examined using disaggregated or uni-level models. For example, Powell et al.’s [27] study found that parental SES was more important in explaining the Hispanic–White BMI gap than the Black–White BMI gap for both genders, whereas neighborhood economic contextual factors were more important in explaining the male BMI gap than the female BMI gap for both Black–White and Hispanic–White ethnic disparities [27]. Krist et al. [28] found that lower neighborhood, but not individual, SES was associated with higher physical activity; however, after considering school SES, associations were attenuated and became insignificant for the relationship between neighborhood SES and physical activity. Uni-level studies provide a good foundation for the current study. The current study aims to answer three questions:

RQ1: Are individual SES, school SES, and neighborhood SES associated with BMI status among Hispanic pre-K students in South Texas?
RQ2: Do individual SES, school SES, and neighborhood SES interact to have an effect on BMI status among Hispanic pre-K students in South Texas?

The prevalence of cross-classified data structures in studies using educational settings (student nested in schools and teachers/classrooms/grades/schools) has continued to increase since the 1990s [29]; however, Ye and Daniel [29] found that only 36 studies in educational settings published between 1994 and 2014 utilized multilevel, cross-classified approaches. This demonstrates the underutilization of cross-classified approaches historically. Cross-classified approaches are not only appropriate to use to properly account for cross-classified data structures, but they also add rigor to the analyses [29]. The authors of the present study conducted an extensive literature search for studies using cross-classified models to examine BMI status in early childhood, and no such studies exist to the authors’ knowledge. Additionally, only one study in this area, mentioned above [26], has utilized multilevel methods. Thus, the use of multilevel, cross-classified models is not only appropriate and rigorous, but it also contributes to the literature as these methods are underutilized, especially in this area of research. This study uses an innovative multilevel, cross-classified model approach (students clustered in schools and neighborhoods) to understand which level or levels of SES affect BMI among a Hispanic sample of pre-K students, as well as how the levels may interact.

## 2. Materials and Methods

### 2.1. Sample

Student data were obtained from a larger quasi-experimental trial of a multicomponent intervention to promote social-emotional learning in a school district in South Texas. Caregivers responded on behalf of pre-K students in the fall of 2019, i.e., baseline (*N* = 269). The analytic sample only included students whose caregivers identified as being Hispanic/Latino (*n* = 237, 89%). The Institutional Review Board at The University of Texas at Austin approved the study protocols and procedures.

### 2.2. Measures

#### 2.2.1. Sociodemographic Characteristics

Individual-level characteristics included gender, caregiver marital status, caregiver education level, caregiver relationship to the child, and family income. These sociodemographic individual-level variables were included as controls, except for family income which was the main exposure variable. Family income was expressed as the income-to-needs ratio, which represents a yearly income dollar amount per person in the household.

#### 2.2.2. Dependent Variable

The dependent variable was the child’s body mass index z-score (BMIz). For population-based assessment, particularly with young children, the z-score is recognized as the best system for analysis because it considers age and sex, which affect developmental growth [30]. The computation requires the child’s biological sex, height in centimeters, weight in kilograms, and age in months. Child height and weight were measured by the school nurse using a standardized scale and stadiometer. Typically, a z-score of 0 is the same as a 50th percentile, a z-score of ±1.0 plots at around the 15th or 85th percentiles, respectively, and a z-score of ±2 plots at roughly the 3rd or 97th percentiles, respectively [31]. BMIz scores were computed using a SAS program written by the Centers for Disease Control and Prevention [32].

#### 2.2.3. Level-Two Measures

There were two level-two variables, which were the other two main exposure variables. The first was neighborhood poverty, expressed as the percent of residents below 100% federal poverty line in the census tract of residence. Neighborhood data were linked to the student data based on census tract geocodes derived from respondent-provided residential addresses. Census tracts were used as approximations of neighborhoods, consistent with past studies [33,34]. Census tract poverty rates were captured from the U.S. Census Bureau’s 2018 American Community Survey 5-Year Estimates [35].

The second level-two variable was school-level SES, derived from aggregate individual-level (family income) data. This variable represents the mean family income-to-needs ratio for the sample from each school. A common method of measuring school SES is the percentage of students receiving free or reduced-price lunch [36,37]; however, all the schools in this sample provide free breakfast and lunch to all students. Thus, there would be no variation across schools using that measure to operationalize school SES.

### 2.3. Statistical Analysis

First, we inspected variables for missing values and normality. We grand-mean centered family income, school SES, neighborhood poverty, and education. We used multiple imputations to handle missing data [38]. The multiple imputation method chosen was the fully conditional method (FCS) using SAS PROC MI. The FCS method involves including auxiliary variables to strengthen the accuracy of the imputation and provides less biased estimates with a smaller sample size and a high fraction of missing data compared to other methods [39]. Twenty imputations were conducted as recommended by the imputation literature [40].

We examined the prevalence of overweight and obesity, respectively, as well as the overall sample characteristics and compared the means to the raw data. We estimated three sets of null or random intercept-only multilevel models: a school-only model, a neighborhood-only model, and a cross-classified multilevel model. These null models allowed us to partition the variance in BMI z-scores into within and between components and to estimate intraclass correlation coefficients (ICCs). The three combined equations for the three null models were:

Schools: Y_ij_ = Υ_00_ + u_0j_ + r_ij_;
Neighborhoods: Y_ik_ = Υ_00_ + v_0k_ + r_ik_;
Schools and Neighborhoods: Y_i(jk)_ = Υ_00_ + u_0j_ + v_0k_ + r_i(jk)_.

The coefficients are represented in the null models, where Υ_00_ refers to the overall mean outcome y across schools and neighborhoods, u_0j_ refers to the random effect for schools, v_0k_ refers to the random effect for neighborhoods, and r_i(jk)_ refers to the random effect for the combination of *j*-th school and *k*-th neighborhood. The null model analyses were conducted using SAS PROC MIXED. The formula to calculate the ICC is: (τ_00_/(τ_00_ + σ^2^)). In this equation, τ_00_ = variance of the level-2 residuals (u) and σ^2^ = variance of the level-1 residuals (r). The mean ICC for schools was 0.28. For neighborhoods, it was 0.06. For both combined, it was 0.27. There was much more variance in BMI z-scores between schools than between neighborhoods, but overall there was significant variance to be explained at each level, which confirmed the use of the multilevel cross-classified model building.

We employed a bottom-up approach for model building, as recommended by Hox [41]. We conducted separate fixed-effect models for all five level-1 variables (gender, caregiver’s marital status, caregiver’s education level, caregiver’s relationship to child, and family income), then moved on to the level-2 variables. Each model was examined for significance to be included in the combined fixed-effect model. A *p*-value of less than 0.25 was set as the criterion for inclusion in the models [42,43]. We used the PROC MIANALYZE procedure after using PROC MIXED to compute one set of fixed effects for all 20 imputations. Next, we assessed whether the explanatory variables at level 1 had a random slope between either of the level 2 contexts. Each random slope model included the explanatory level 1 variable at the school level and neighborhood level, respectively. We added cross-level interactions between explanatory level-2 variables and explanatory level-1 variables that had significant slope variation in the random-effects models. This involved separate models examining interactions between schools and neighborhoods individually. Lastly, model fit was tested by examining the change in the negative two log-likelihood (-2LL) between models via a chi-square test [41]. The restricted maximum likelihood method was used for these tests. Lastly, a working model was tested against the level-1 covariate model. The maximum likelihood method was used for this test since the models only differed in fixed effects. The final model was decided with consideration of model fit and model parsimony. All analyses were conducted using SAS software version 9.4 for all analyses [44].

## 3. Results

The students represented thirty-two neighborhoods (i.e., census tracts) and twelve public elementary schools. The mean number of pre-K students in each school was 19.75 (range: 12–26), and 90% of schools had at least sixteen students. The mean number of pre-K students in each neighborhood was 7.41 (range: 1–22), and 50% of neighborhoods had at least five students from the sample. Table 1 presents means and SDs or frequencies and percentages for all individual-, school-, and neighborhood-level variables included in analyses. The sample had a slightly higher proportion of girls (54%) compared to boys (46%) and consisted largely of caregivers who were the child’s biological or adoptive parent (93%). The sample also consisted of a slightly higher proportion of caregivers who were married or living with a partner (58%) compared to caregivers who were unmarried or not living with a partner (42%). The average number of years of schooling completed by caregivers was 12 (standard deviation (*SD*) = 2.52). The average family income-to-needs ratio was USD 6273 (*SD* = 6510). The average school SES was USD 6430 (*SD* = 1695). For example, on average, a family of five people would have a combined yearly income of around USD 32,000. School-level SES ranged from USD 3567.46 to USD 8808.11. The average neighborhood poverty rate was 7.8% (*SD* = 1.78), which was well below what is considered “high poverty” (20%) [45]. Neighborhood poverty rates ranged from 2% to 13.5%. The mean BMI z-score was 0.49, within the healthy weight range, and 24% of the sample was classified as overweight, with an additional 5% classified as obese.

Table 2 presents results from the cross-classified mixed-effects linear regression models that examined the unique associations of individual-, neighborhood- and school-level variables with individual student BMI z-scores. After examining the individual unadjusted models, family income was the only variable that did not meet the *p* < 0.25 threshold for inclusion in the models as fixed effects. In the combined level-1 covariate model that excluded family income, all level-1 variables remained significant for inclusion at *p* < 0.25. The model building resulted in four significant random-slope estimates (not shown in Table 2). Random slopes for family income and caregiver relationship to the child were significant both at the neighborhood and school levels. Four cross-level interactions between schools and neighborhoods and family income or caregiver relationship to the child were tested. None of the cross-level interaction models were significant (not shown in Table 2). The model fit statistics of a working model, which included all study variables except family income, were tested individually against all four significant random slope models, and no significant differences were found. The working model was then tested against the covariate model, and the chi-square test was significant at *p* = 0.022. This indicated that the working model with all study variables except family income was the best fitting and “final” model.

The final model resulted in a significant association between caregiver relationship to the child and BMI z-score (*B* = −1.11, *SE* = 0.38; *p* < 0.05), such that students’ BMI z-score was approximately 1.11 points lower for those whose caregiver was not their biological/adoptive parent, compared to those whose caregiver was their biological/adoptive parent, controlling for the other variables. Child gender, caregiver marital status, and caregiver education had no significant main effects on BMI z-score in the final model.

Both level-2 variables had a significant main effect on BMI z-score in the final model. We found a significant association between school SES and BMI z-score (*B* = −0.13; *SE* = 0.06; *p* < 0.05), such that individual students’ BMI z-score decreased by approximately 0.13 points for each one-thousand dollar increase in the school SES, controlling for the other variables. We also found a significant association between neighborhood poverty and BMI z-score (*B* = −1.41; *SE* = 0.49; *p* < 0.01), such that individual students’ BMI z-score decreased by approximately 1.41 points for each 1-point increase in neighborhood poverty, controlling for the other variables.

The total explained variance for the school level was 0.30, and the total explained variance for the neighborhood level was 0.09. For the school level, we explained 30% of the 28% in total to be explained. For the neighborhood level, we explained 9% of the 6% in total to be explained. Adding variables to the final model attenuated the between-level variance for schools (0.48 to 0.40) and neighborhoods (0.32 to 0.24), but the individual-level residual variance increased (1.09 to 1.15).

## 4. Discussion

Two of the study’s main findings provide insights into how various levels of SES affect child weight status. The final model did not include cross-level interactions or random slopes; however, the evidence indicates that school SES and neighborhood poverty affect child weight status in differing ways. Consistent with the previous literature [46], students’ BMI z-score decreased as school SES increased. For neighborhood poverty, students’ BMI z-score decreased as neighborhood poverty increased. While this contrasts with our initial hypothesis, this result is not unprecedented in the literature [25,28]. The third significant finding was that students’ BMI z-score was lower for those whose caregiver was not their biological/adoptive parent compared to those whose caregiver was their biological/adoptive parent. This finding was also unexpected and required further examination, given the paucity of the literature examining this relationship.

Given that the school SES variable was the mean family income of the sample from each school, it is not surprising that this finding aligns with the findings from studies focusing on individual/family SES (i.e., an inverse relationship between SES and obesity). This finding also adds to the evidence from studies utilizing other measures of school SES, such as public/private status and percent of students on free or reduced-price lunch, pointing to the disparity between schools with lower SES and schools with higher SES that could continue to widen over time. Importantly, pre-Kindergarten may be a critical period to implement interventions designed to target widening obesity disparities. The literature continues to suggest that obesity disparities should be addressed as early in life as possible to prevent larger disparities in adolescence and even adulthood [47,48,49]. This lends evidence to the appropriateness of the larger longitudinal intervention programming being implemented in congruence with this cross-sectional study. Future longitudinal research should examine if school SES remains protective as kids age and experience exposure to their peers and the school nutrition program. It is possible that being in a pre-Kindergarten cohort with a higher SES was protective of BMI z-score because a cohort with a higher mean SES may positively influence each other with similar, healthier habits compared to a cohort with a lower mean SES.

The association between increased neighborhood poverty status with decreased individual BMI z-scores was unexpected, given that most of the prior literature documents that individuals from poorer neighborhoods engage in less healthy lifestyles [19,20]. However, studies that found similar results to the present study offer insights into this finding. Kimbro et al. [25] and Krist et al. [28] suggest that children from lower SES neighborhoods have more unstructured time that they fill with time playing outside and being physically active (i.e., expending calories). On the other hand, the literature suggests children from lower SES neighborhoods have fewer safe places to play and less green space to play compared to children from higher SES neighborhoods [11,26]. Notably, however, children may engage in high levels of physical activity even in neighborhoods that lack safe spaces or are perceived as unsafe by parents [25,50]. Further studies should examine children’s own perceptions of outdoor spaces for physical activity since these could be protective against overweight/obesity. This sample may be too young to engage in unstructured playtime on their own, but perhaps children from lower SES neighborhoods are attending schools closer to their homes, in which they may be expending calories by walking to school instead of being driven to school. Another important factor to consider for this finding is energy intake. The existing literature does not support the findings from the present study. For example, Leung et al. [51] found neighborhood food and retail availability may be inversely associated with young girls’ energy intakes. Additionally, Keita et al. [52] found that children in disadvantaged neighborhoods had poorer diet quality and consumed more calories. Alternatively, Mayne et al. [53] found higher perceived neighborhood safety and collective efficacy were associated with a higher daily intake of fruits/vegetables. Further research is needed to understand why certain studies report an inverse relationship between neighborhood poverty and obesity in childhood.

It is worth discussing the findings related to family income. The fixed effect was not significant, but the random effects were significant. This indicates that family income operated differently for each family—in both positive and negative directions—at both the school and neighborhood levels. A deeper examination into how family income interacts with school SES and neighborhood poverty may reveal how family income is protective for families in varying schools and neighborhoods. For example, further analyses may reveal that higher school SES is only associated with healthier BMIz for children with higher family income compared to children with lower family income. Alternatively, it may reveal that higher neighborhood poverty is only associated with healthier BMIz for children with higher family income compared to children with lower family income.

The finding that students’ BMI z-score was lower for those whose caregiver was not their biological/adoptive parent when taken in the context of this sample indicates a need to focus on grandparents as primary caregivers since they were the second-largest subgroup of caregiver respondents. Grandparents caring for grandchildren either part- or full-time has increased significantly since the 1990s, and this remains true among Hispanic families [54,55]. The literature on the role of grandparents and caregiving related to obesity offers insights into this unexpected finding. The literature consistently reports the positive impact grandparents who are primary caregivers have on their grandchildren [56,57,58]. Some of the reasons why grandparents have a positive impact are that grandparents report restricting child intake more than parents and provide healthier options than parents [56,57]. For Hispanic grandparents specifically, positive impacts may have resulted from doing physical activity with grandchildren, taking grandchildren to places for physical activities, and rewarding grandchildren for doing physical activities [55]. Whereas children from lower SES neighborhoods may be receiving more playtime due to unstructured time, children with grandparents as caregivers may be receiving a lot of physical activity from more structured playtime with grandparents.

Several strengths and limitations should be noted. One strength is the objective nature of the height and weight scores collected by the school nurses to calculate BMI. Limitations in this study include missing data for income and height and weight. Maternal BMI and maternal age are important predictors of child BMI; however, since not all the caregivers were the child’s mother, we decided not to include these variables. The sample was largely low-income and homogenous in regard to family income; thus, this may have affected the ability to detect differences across family income. It should also be noted that aggregating family income to develop the school SES variable is not ideal since it is based on a sample of the pre-Kindergarten families instead of all the families at the school. Additionally, the sample was not overly diverse in regard to neighborhood poverty, and greater variation could have produced larger effect sizes. This is not a nationally representative sample but may represent Hispanic Pre-Kindergarten students in South Texas due to the demographic characteristics of the sample and the population in South Texas.

## 5. Conclusions

It is troubling that 30% of children in this sample fall within the overweight/obese categories. This finding provides evidence for the continued need for obesity treatment and prevention studies, especially in early childhood populations. The results suggest that neighborhood SES and school SES both play a role in the development of obesity in early childhood. Future studies should further investigate why higher neighborhood poverty may be resulting in lower weight status and at what point this trajectory changes, given that the literature has documented higher neighborhood poverty is associated with higher adult BMI in the United States [58]. Additionally, future studies with a larger, more diverse sample in regard to family income may be able to detect interactions between family-level income and school or neighborhood SES. Interactions may provide insights into how contextual factors interact to protect children from obesity in early childhood. Addressing this issue is critical at this age, as it could help reduce negative outcomes into adulthood.

## Figures and Tables

**Table 1:** Descriptive Characteristics of All Study Variables, *n* = 237.

	*n*/*M*	%/*SD*
Child’s Sex (Girl)	128	54%
Caregiver’s Relationship to Child (Parent)	214	93%
Caregiver Marital Status (Married)	132	58%
Caregiver Education (# years completed)	12	2.52
Family Income (USD amount per person/yearly income)	6273	6510
School Socioeconomic Status (average family income for each school)	6430	1695
Neighborhood Poverty (% below poverty line)	7.80 Range: 2–13.5	1.78
Child’s Body Mass Index *z*-score	0.49	1.27
Overweight	41	24%
Obese	9	5%

*Note. M* = mean. *SD* = standard deviation. Income-to-needs ratio: household income/number of people in the household. Family income is expressed as an income-to-needs ratio that represents the dollar amount of yearly income per person; considering the average is USD6430, a family of five people would have a combined yearly income of around USD32,000. School socioeconomic status = average family income-to-needs ratio for each school.

**Table 2:** Cross-Classified Mixed Effects Linear Regression for BMIz.

	Unadjusted Models *B* (*SE*)	Covariate Model *B* (*SE*)	Final Model *B* (*SE*)
*Main Effects*			
Girl	−0.27 (0.18) ++	−0.22 (0.18) ++	−0.10 (0.18)
Non-parent	−1.03 (0.39) ++	−1.00 (0.38) ++	−1.11 (0.38) *
Unmarried	0.42 (0.18) ++	0.43 (0.18) ++	0.34 (0.18)
Education	−0.06 (0.04) ++	−0.06 (0.04) ++	−0.07 (0.03)
Family Income	−0.02 (0.02)		
School Socioeconomic Status	−0.14 (0.06) ++		−0.13 (0.06) *
Neighborhood Poverty	−0.10 (0.51) ++		−0.14 (0.49) **
*Random Effects*			
Level-2 Intercept (School)	-	0.48	0.40
Level-2 Intercept (Neighborhood)	-	0.32	0.24
Residual	-	1.09	1.15
*Model Fit*			
−2LL	-	774.04	764.39
AIC	-	778.04	768.06
BIC	-	774.04	764.39
χ^2^ test between Covariate and Final model	-	*p* = 0.022	

*Note.* Reference groups: Sex = Boy, Primary Caregiver = Parent, Marital Status = Married. *B* = main effect. *SE* = standard error. Family income is expressed as an income-to-needs ratio that represents the dollar amount of yearly income per person; considering the average is USD 6430, a family of five people would have a combined yearly income of around USD 32,000. School socioeconomic status = average family income-to-needs ratio for each school. * *p* < 0.05. ** *p* < 0.01. ++ *p* < 0.25, covariate inclusion threshold.

## Data Availability

The data presented in this study are available on request from the corresponding author. The data are not publicly available due to the study’s ongoing status.
